# Predictors of long-term response to abiraterone in patients with metastastic castration-resistant prostate cancer: a retrospective cohort study

**DOI:** 10.18632/oncotarget.9485

**Published:** 2016-05-19

**Authors:** Elena Verzoni, Ugo De Giorgi, Lisa Derosa, Orazio Caffo, Francesco Boccardo, Gaetano Facchini, Luca Porcu, Fabio De Vincenzo, Alberto Zaniboni, Vincenzo Emanuele Chiuri, Lucia Fratino, Daniele Santini, Vincenzo Adamo, Rocco De Vivo, Angelo Dinota, Caterina Messina, Riccardo Ricotta, Claudia Caserta, Claudio Scavelli, Marina Susi, Alfredo Tartarone, Giuseppe Surace, Alessandra Mosca, Michele Bruno, Sandro Barni, Paolo Grassi, Giuseppe Procopio

**Affiliations:** ^1^ Unit of Medical Oncology 1, Fondazione IRCCS Istituto Nazionale Tumori, Milan, Italy; ^2^ Department of Medical Oncology, IRST, IRCCS, Meldola, Italy; ^3^ Unit of Medical Oncology 2, Istituto Toscano Tumori, Pisa, Italy; ^4^ Ospedale Santa Chiara, Trento, Italy; ^5^ IRCCS AOU San Martino IST and University of Genoa, Italy; ^6^ Unit of Medical Oncology, Department of Uro-Gynecological Oncology, Istituto Nazionale Tumori, Fondazione G. Pascale IRCCS, Naples, Italy; ^7^ Department of Oncology, IRCCS- Istituto di Ricerche Farmacologiche Mario Negri, Milan, Italy; ^8^ Istituto Clinico Humanitas, Rozzano, Italy; ^9^ Fondazione Poliambulanza, Brescia, Italy; ^10^ Ospedale Vito Fazzi, Lecce, Italy; ^11^ Istituto Nazionale Tumori CRO, Aviano, Italy; ^12^ Policlinico Universitario Campus Biomedico, Roma, Italy; ^13^ Medical Oncology Unit, AO Papardo, Messina, Italy; ^14^ Ospedale San Bortolo, Vicenza, Italy; ^15^ Ospedale San Carlo, Potenza, Italy; ^16^ Azienda Ospedaliera Papa Giovanni XXIII, Bergamo, Italy; ^17^ Niguarda Cancer Center, Ospedale Niguarda Ca' Granda, Milan, Italy; ^18^ AO Santa Maria, Terni, Italy; ^19^ Ospedale S. Cuore di Gesù, Gallipoli, Italy; ^20^ Ospedale Madonna delle Grazie, Matera, Italy; ^21^ IRCCS Centro di Riferimento Oncologico della Basilicata (CROB), Rionero in Vulture, Italy; ^22^ Ospedale D. Camberlingo, Francavilla Fontana, Italy; ^23^ AOU Maggiore della Carità, Novara, Italy; ^24^ PO San G. Moscati, ASL Taranto, Italy; ^25^ AO Treviglio, Italy

**Keywords:** abiraterone acetate, castration-resistant, predictive factors, prostate cancer, retrospective studies

## Abstract

We aimed to identify clinical predictors of long-term response to abiraterone (defined as >12 months drug exposure) in a retrospective cohort of metastatic castration-resistant prostate cancer patients treated in post-docetaxel setting at 24 Italian centers. The Cox proportional hazards model was used to analyze the association between clinical features and the duration of drug exposure. Results were expressed as hazard ratios (HR) with associated 95% confidence intervals (CI). A total of 143 patients met the inclusion criteria. Their median age was 73 years, median Gleason score 8 and median abiraterone exposure 20 months. At the univariate analysis, a significant correlation with the duration of abiraterone exposure was found for Gleason score (HR 0.82, 95% CI 0.71-0.96; p=0.012), PSA (HR 1.10, 95% CI 1.03-1.18; p=0.08) and lactic dehydrogenase levels (HR 1.22, 95% CI 1.02-1.46; p=0.027), while the association between lower alkaline phosphatase levels and treatment duration was marginally significant (HR 1.07, 95% CI 0.99-1.16; p=0.074). Only PSA and Gleason score were predictive of long-term treatment duration in the multivariate analysis. No other clinical factors resulted to be predictive of sustained response to abiraterone, including metastatic disease at diagnosis and visceral disease, suggesting that all subgroups of patients may derive a substantial clinical benefit from abiraterone treatment. These findings need to be validated in prospective, larger studies.

## INTRODUCTION

Treatment options for metastatic castration-resistant prostate cancer (mCRPC) have expanded considerably over the last few years, but how to select the most appropriate drug for each patient in order to obtain maximum benefit from the available therapies still remains unclear. Although recent studies have shown that molecular biomarkers such as androgen receptor splice variant-7 (AR-V7) or ERG gene status hold promise as potential predictors of response [1–3], at present there are no validated predictive biomarkers able to inform clinicians on best treatment selection for mCRPC patients [4]. Therefore, in clinical practice treatment strategies are decided empirically by taking into account mainly patient- and disease-related characteristics, as well as the risk-benefit ratio and the safety profile of the available drugs. AR axis-targeted therapies have become the mainstay of treatment in metastatic CRPC, both in chemotherapy-naïve patients and in those progressing after docetaxel. Abiraterone, a selective inhibitor of CYP17, was the first available drug in this class and has been widely used in Europe since its approval in 2011. In the registration trial COU-AA-301, treatment with abiraterone plus prednisone significantly increased median overall survival compared with placebo (15.8 vs 11.2 months, HR 0.74, 95% CI 0.64-0.86; p<0.0001) in mCRPC patients previously treated with docetaxel, with a median duration of abiraterone exposure of 7.4 months [5].

In this study we retrospectively analyzed clinical records of men with mCRPC who had received abiraterone post-docetaxel in routine clinical settings in Italy, with the aim to identify potential clinical predictors of long-term response to abiraterone, by defining long-term responders as those patients receiving this drug for >12 months.

## RESULTS

We identified 143 patients who met the inclusion criteria and were evaluated. Demographic and clinical characteristics are summarized in Table 1. Median age was 73 years (range 47-87 years) and median Gleason score was 8 (range 3-10). The majority of patients (89%) had bone metastases only, and 28% had synchronous metastases at diagnosis. Median serum prostate-specific antigen (PSA), alkaline phosphatase (ALP) and lactic dehydrogenase (LDH) levels before starting abiraterone were 38 ng/dL (range 0.45-1339 ng/dL), 98.5 U/L (range 36-1778 U/L) and 269.5 U/L (range 113-1119 U/L), respectively. Patients received a median of 3 prior lines (interquartile range [IQR] 2-4) of therapy before starting abiraterone, including chemotherapy (86%). The main reasons for treatment failure before starting abiraterone included PSA progression (33%), both PSA andradiological progression (55%) and radiological progression only (12%).

**Table 1:** Demographic and clinical characteristics of the study cohort

	N (%)	Median	IQR	Range
Patients	143			
Age	143	73.2		47.3-87.2
Stage at diagnosis				
*M0*	80 (55.9)			
*M1*	40 (28.0)			
*Missing data*	23 (16.1)			
Gleason score	129	8		6-10
Metastatic sites^#^				
*Bone only*	127 (88.8)			
*Bone and/or other organs*	12 (8.4)			
*Missing data*	4 (2.8)			
PSA (ng/dL)	141	38		0.45-1339
ALP (U/L)	108	98.5		36-1778
LDH (U/L)	102	269.5		113-1119
Abiraterone treatment				
*Duration (months)*		19.8	14.3-29.4	12.0-49.0^§^
*Terminated*	109 (76.2%)			
*Ongoing*	34 (23.8%)			
Previous therapies				
*No. of lines*	143	3	2-4	1-6
*Hormonal therapy*	18 (12.6%)			
*Chemotherapy*	123 (86.0%)			
*Missing data*	2 (1.4%)			

* At the time of analysis;

# At initiation of abiraterone treatment;

§ Ongoing

PSA=Prostate-specific antigen; ALP=alkaline phosphatase; LDH=Lactic dehydrogenase

**Figure 1:**
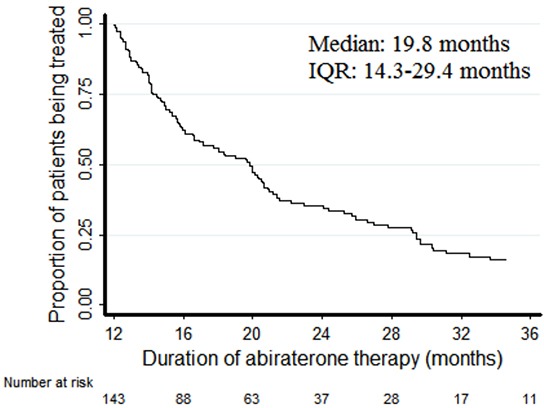
Kaplan-Meier plots of duration of treatment with abiraterone

Median follow-up was 34.6 months and median duration of abiraterone treatment was 19.8 months (IQR 14.3-29.4 months) (Figure 1). At the time of analysis, 34 (24%) patients were still receiving abiraterone. In the remaining patients (72%) treatment was discontinued due to disease progression, while 31 (23%) had died. The treatment was well tolerated. The only adverse events which were reported were grade 1 (G1) hypokalemia (5%), G1-G2 asthenia (17%; G1, 14% and G2, 3%), G2 anemia (5%), G1 mucositis (5%) and G1 diarrhea (3%). No G3-G4 adverse events were reported.

PSA response ≥50% was observed in 80% of patients (n=114), with a disease control rate of 88% (n=126).

**Figure 2:**
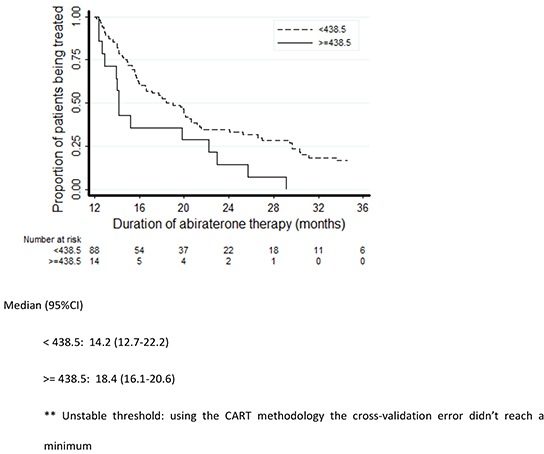
Kaplan-Meier plots of duration of treatment with abiraterone according to LDH levels

**Figure 3:**
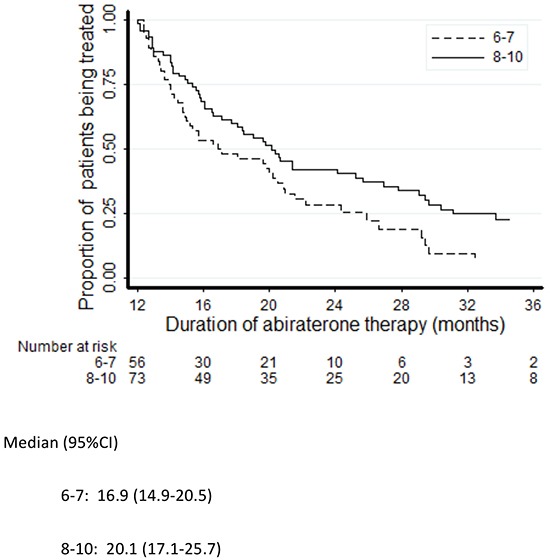
Kaplan-Meier plots of duration of treatment with abiraterone according to Gleason score

**Figure 4:**
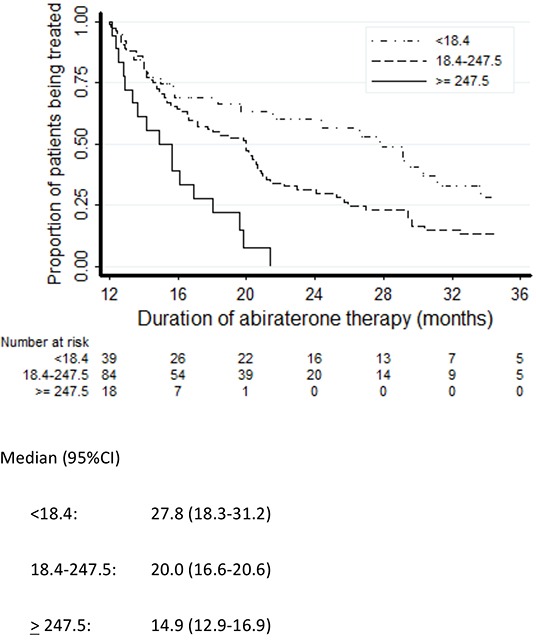
Kaplan-Meier plots of duration of treatment with abiraterone according to PSA levels

**Figure 5:**
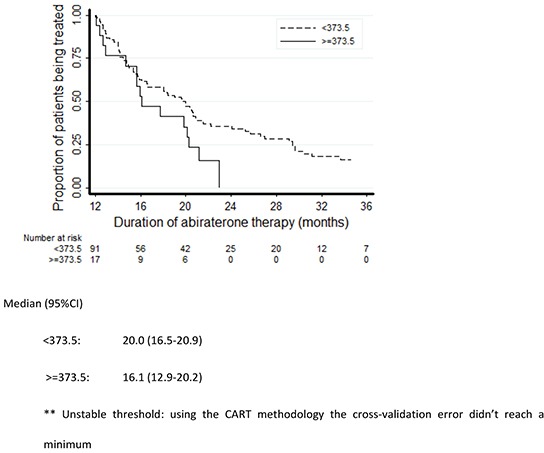
Kaplan-Meier plots of duration of treatment with abiraterone according to ALP levels

Three predictors were significantly associated with the duration of abiraterone treatment: Gleason score (hazard ratio [HR] 0.82, 95% CI 0.71-0.96, *p*=0.012), PSA (the relative hazard function was time varying as reported in Table 2, *p*=0.001; unit=100 ng/dL) and LDH (HR 1.22, 95% CI 1.02-1.46, *p*=0.027; unit=100 U/L). The association between ALP and abiraterone exposure did not quite reach statistical significance (HR 1.07, 95% CI 0.99-1.16; *p*=0.074; unit=100 U/L). While increasing levels of circulating tumor biomarkers (PSA, ALP and LDH) were associated with increased risk of abiraterone interruption, a high Gleason score was found to predict decreased risk of progression. No statistically significant association with the duration of exposure to abiraterone emerged for age, M-staging at diagnosis (M0 vs M1) or site of metastases (bone or visceral) (Table 2). The Kaplan-Meier plots of abiraterone treatment duration according to LDH, Gleason score, PSA and ALP are reported in Figures 2–5. In the multivariate analysis only Gleason score and PSA were independent predictors of abiraterone treatment duration (Table 3).

**Table 2:** Univariate analysis of duration of treatment with abiraterone

Parameter	HR point estimate	95% CI	p-value
*Continuous variables*			
Age (years)^*^	1.05	0.81-1.37	0.722
Gleason score	0.82	0.71-0.96	0.012
PSA^#^	1.00	0.89-1.13	0.001
*Linear interaction term with time*°	1.03	1.01-1.06	
ALP^#^	1.07	0.99-1.16	0.074
LDH^#^	1.22	1.02-1.46	0.027
*Categorical variables*			
Staging at diagnosis			
M0	1		
M1	0.92	0.59-1.45	0.733
Metastatic sites			
Bone only	1		
Other	0.80	0.40-1.59	0.526

° Time 0 in the Cox model: 12 months

* Relative risk of terminating treatment for each 10 years of age;

# Unit divided by 100

PSA=prostate-specific antigen; ALP=alkaline phosphatase; LDH=lactic dehydrogenase

**Table 3:** Multivariate analysis of duration of treatment with abiraterone^†^

Parameter	HR point estimate	95% CI	p-value
Gleason score	0.84	0.72-0.98	0.029
PSA^#^	0.96	0.84-1.10	0.006
*Linear interaction term with time*°	1.04	1.01-1.07	

† Gleason score, PSA, ALP, LDH were introduced as candidate predictors in the backward selection Cox model

° Time 0 in the Cox model: 12 months

# Unit divided by 100

PSA=prostate-specific antigen; ALP=alkaline phosphatase; LDH=lactic dehydrogenase

## DISCUSSION

The most significant finding of this study is the considerable proportion of heavily pre-treated mCRPC patients who achieved, in a routine oncology practice setting, a relevant clinical benefit with abiraterone. The duration of response exceeded, in many cases, two years (median 20 months). This long-term response was observed also in patients with unfavorable prognostic features such as high Gleason score, synchronous metastases at diagnosis or visceral disease.

A feature that sets our study apart from other similar investigations is the fact that we looked for potential clinical predictors of long-term response to abiraterone by focusing on a cohort of patients with a duration of drug exposure longer than expected (as compared with published data), rather than searching for factors that might help discriminate between responders and non/poor responders. The cut-off point of 12 months for definition of long-term response was arbitrarily selected based on the median abiraterone exposure of 7.4 months (range 0.2-25.6) reported in the pivotal abiraterone COU-AA-301 study in chemotherapy-treated patients [5].

A significant association with the duration of drug exposure was found for Gleason score, PSA and LDH levels before starting treatment, with a marginally significant association for ALP. The correlation between rising levels of circulating tumor biomarkers and poorer response were not surprising, given that PSA, LDH and ALP are known prognostic factors for survival in CRPC across a wide range of therapies, or in the absence of life-prolonging treatments [6–9]. Unexpectedly, a higher Gleason score was found to correlate to longer drug exposure. This association, however, has been documented in other studies, where high Gleason score was found to be predictive of sustained treatment response in docetaxel trials [10, 11] but not in the “new hormonal compounds” trials. In a recent retrospective analysis of the COU-AA-301 and COU-AA-302 trials by Fizazi et al [12], initial Gleason score was not predictive of response to abiraterone in either chemotherapy naïve or post-docetaxel patients, as no significant difference in treatment benefits was found between patients with a Gleason score of < 8 versus ≥ 8.

No other potential predictors of long-term response emerged from our analysis. In particular, the presence of synchronous metastases at diagnosis and visceral disease were not associated with a less favorable outcome (although only 8.4% of patients had visceral metastases in our cohort). The lack of a significant association between presence of visceral metastases and radiological progression or survival has been observed in other recent retrospective studies in patients treated with AR-targeted therapies [13, 14]. These findings are in keeping with the results of subgroup and exploratory post-hoc analyses of the COU-AA-301 trial and suggest that all subgroups of patients are likely to derive at least some clinical benefit (not excluding the possibility of a long-term benefit) from abiraterone treatment, irrespective of unfavorable baseline clinical characteristics [5, 15]. Furthermore, most of our patients had skeletal metastases only, and they might well represent ideal candidates for abiraterone treatment, as suggested by recent data documenting direct bone anabolic and anti-resorptive effects of this drug both *in vitro* and in CRPC patients [16].

Our study had limitations that need to be taken into account when interpreting the results, including the retrospective design and the relatively small patient population. The lack of a control group of early refractory patients did not allow to discriminate between early refractory and long responding patients. Moreover, we evaluated outcome based on abiraterone exposure time rather than using standardized outcome measures such as overall survival (OS) or progression-free survival (PFS).

Several biomarkers have been recently proposed as potential predictors of treatment response in mCRPC, including tumor-associated genetic profiling parameters from biopsies, circulating biomarkers, imaging data and clinical variables. AR-V7 has been linked to primary resistance to either abiraterone or enzalutamide, and monitoring AR-V7 status in circulating tumor cells (CTCs) over the course of treatment may predict sensitivity or resistance to AR-targeted agents [1, 2]. Analysis of AR-modulated ERG expression in archival tumor biopsies from patients enrolled in the COU-AA-302 trial showed that ERG gene fusion expression (2^+^ Edel cancers), previously considered as unfavorable prognostic factor for survival, was associated with the greatest clinical benefit from abiraterone treatment [17]. Imaging biomarkers may also assist in therapeutic monitoring and outcome prediction, as suggested by the results of a pilot study on the use of 18-F fluorocholine positron emission tomography/computed tomography (FCH-PET/CT) in early evaluation of abiraterone response in the post-docetaxel setting [18]. Nevertheless, the lack of standardization, the limited availability and the cost of the newer imaging modalities are still an issue. Among circulating biomarkers, CTCs and neutrophil/lymphocyte ratio (NLR, a manifestation of tumor-promoting inflammation) have been shown to be prognostic in many cancer types and are receiving much attention in the context of mCRPC [6, 19]. A biomarker panel based on CTCs count and LDH was shown to meet the Prentice criteria as a surrogate for OS in the COU-AA-301 trial, and validation studies are ongoing [20]. On the other hand, preliminary data suggest that a 30% decline in CTCs levels 4 weeks after treatment initiation from baseline may predict treatment response to abiraterone or chemotherapy in patients with mCRPC [21]. In a cohort of mCRPC patients treated with abiraterone in a Canadian study (53% post-docetaxel), Leibowitz-Amit et al. identified a composite score derived from the sum of baseline NLR and the extent of metastatic spread as a predictor of PSA response [22]. Preliminary data have also shown that an increase in NLR persisting during enzalutamide treatment in chemotherapy-treated mCRPC patients may predict a poor response to enzalutamide [23].

Retrospective analysis of the Temporary Authorization for Use programme in France showed that PSA decrease at 3 months was a predictive factor for abiraterone treatment duration [24]. In our experience we could not explore this correlation because of different timepoints for PSA evaluation. Among clinical biomarkers, the duration of previous androgen-deprivation therapy (ADT) response, or time to CRPC, has received much attention as a potential predictor of response to the new AR-targeted therapies. In a retrospective study in 61 mCRPC patients treated with abiraterone post-docetaxel, Ashfar et al. identified three independent predictors of OS: hemoglobin levels, ECOG-PS at starting abiraterone, and duration of response to primary androgen-deprivation therapy [13]. Similarly, time to CRPC ≥12 months and ECOG-PS score 0-1 were associated with improved PFS in a cohort of 173 patients treated with enzalutamide, abiraterone or other hormonal therapies at two French cancer centers [14]. The duration of previous hormonal therapy resulted to be associated with better OS also in the abiraterone-treated cohort (n=306) of the aforementioned Temporary Authorization for Use study [24]. Furthermore, time to development of CRPC was identified as the strongest predictor of PSA response, PSA-PFS and OS also when AR-targeted therapies were used in a second line setting, as reported in a retrospective study evaluating the outcome of 126 patients treated with sequential abiraterone and enzalutamide [25]. Other studies, however, failed to identify a significant association between duration of previous androgen-deprivation therapy and response to subsequent AR-targeted treatments (including the cited investigation by Leibowitz-Amit et al.) [22]. In our study, data about the duration of previous hormonal treatment were not always available for analysis and therefore we could not explore this factor.

It should be emphasized that none of the biomarkers so far investigated have yet been validated as predictors of treatment response [4, 6].

In conclusion, our retrospective analysis in patient with mCRPC, mostly treated in post-docetaxel setting, showed that high Gleason score, lower PSA and LDH levels before starting abiraterone were significantly associated with long-term abiraterone exposure (a marginal significance being found for lower ALP). We failed to identify more specific clinical factors predictive of sustained response to this drug, suggesting that all suitable patients may receive abiraterone therapy on the basis of clinical or tumor-related characteristics. Prospective studies in larger patient populations are needed to confirm our findings.

## MATERIALS AND METHODS

Patients with histologically confirmed mCRPC who were treated with abiraterone for >12 months at 24 Italian cancer centers from October 2011 to July 2014 were retrospectively identified. Clinical records were collected and the following demographic and clinical parameters were analyzed: age at initial abiraterone exposure, duration of abiraterone treatment, number and type of previous anticancer therapies, serum levels of PSA, ALP and LDH before starting abiraterone, Gleason score at diagnosis, type of disease progression before starting abiraterone (PSA progression only or radiological progression), M-staging at diagnosis, sites of metastases, PSA response rates and objective response (in men with measurable disease). The study endpoint was the time from the start of abiraterone treatment to abiraterone interruption from any cause. Patients' data were collected for retrospective analysis in April 2015.

### Statistical analysis

Descriptive statistics were used to summarize demographic and clinical features (median, IQR and range for continuous variables, absolute and percentage frequencies for categorical variables). The Cox proportional hazards model was used to detect and estimate the statistical association between the patients' demographic-clinical features and the duration of abiraterone exposure; the hazard ratio (HR) was used as population parameter and the Wald test statistic was used to test H_0_: HR = 0. All statistical tests were two-sided and statistical significance was detected at the 5% probability level (p-value<0.05). For each predictor the assumption of proportional hazard was tested using a Cox's model with a constant and linear term in time. For each continuous predictor associated to abiraterone treatment duration the best thresholds were identified using the CART methodology [26], with regression trees being generated by means of the Recursive Partitioning and Regression Trees (Rpart) package. Each tree was pruned back in order to avoid data overfitting and the tree size that minimized the cross-validated error was chosen. At least one threshold was mandatory. A backward elimination Cox regression procedure at 0.05 level was used to identify the strongest predictors of abiraterone treatment duration.

The Kaplan-Meier method was used to estimate survival functions. The inverse Kaplan-Meier method was used to estimate median follow-up; patients still receiving abiraterone at the time of the last contact were right censored.

Apart from CART methodology, the statistical analysis was performed using the SAS software (SAS Institute, Cary, NC, USA), version 9.2.
